# The Impact of Metachronous Colorectal Neoplasia Requiring Surgery After Cessation of Colonoscopic Surveillance at Age 75

**DOI:** 10.1111/ans.70199

**Published:** 2025-05-30

**Authors:** Ammar Manasawala, John Woodfield, Kari Clifford, Mark Thompson Fawcett

**Affiliations:** ^1^ Department of Surgical Sciences Otago Medical School, University of Otago Dunedin New Zealand

**Keywords:** colorectal surgery, colorectal surveillance, metachronous colorectal neoplasia

## Abstract

**Background:**

To assess the clinical and financial impact of metachronous colorectal neoplasia (MCRN) requiring surgery after cessation of colonoscopic surveillance at age 75.

**Methods:**

The Otago Clinical Audit database was interrogated to identify all colorectal neoplasia (CRN) resections between January 2020 and November 2022 and additional patients undergoing surgery for MCRN aged ≥ 75 (MCRN ≥ 75) between 2010 and 2020. The morbidity, hospital stay, and costs of surgery for MCRN ≥ 75 cases were compared to first colorectal neoplasia (FCRN) cases ≥ 75 and to all other colorectal resections, with and without propensity matching.

**Results:**

MCRN was identified in 3.1% of patients < 75 and 13.1% of patients ≥ 75. Identifying a further 41 patients with surgery for MCRN ≥ 75 after 2010 resulted in 55 patients with MCRN aged ≥ 75, 93 with FCRN aged ≥ 75, and 130 with CRN aged < 75. The median(IQR) age for MCRN ≥ 75 was 81 (78–86). Surgery for MCRN ≥ 75 compared to FCRN ≥ 75 resulted in complication rates of 70.9% and 50.5% (*p* = 0.024), hospital stay nine versus seven days (*p* = 0.012), readmissions 20% versus 6.5% (*p* = 0.026) and cost of NZD 31 021 versus 24 157 (*p* = 0.028). When compared to all other resections, and adjusting for different approaches to rectal cancer in elderly patients, these differences all increased. The estimated annual hospital cost for MCRN ≥ 75 surgery was NZD 317 777.

**Conclusion:**

MCRN accounted for 13.1% of operations in patients aged ≥ 75. This resulted in more morbidity and cost than surgery for FCRN ≥ 75. Stopping surveillance of those with previous surgery for CRN at 75 years of age results in significant institutional cost and patient morbidity.

## Graphical abstract

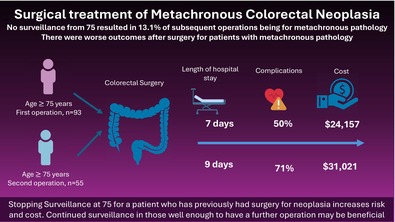

## 1 Introduction

With an incidence of 44.4 in men and 36.9 in women per 100 000, colorectal cancer is the third most common cancer diagnosis and the second highest cause of cancer death in New Zealand [1]. The risk of colorectal cancer increases with age [2]. Due to the increasing population age, the total number of cases of primary and metachronous colorectal neoplasia (CRN) in the elderly is growing [3].

Health New Zealand Southern District has an institutional policy of no surveillance colonoscopy from age 75. While this was set historically in the context of limited resources, it has not been updated. The NZ national guidelines state: ‘People aged 75 or older should be carefully considered before offering surveillance because the potential risks associated with ongoing colonoscopic surveillance are likely to outweigh the benefits’ [4]. As colorectal surgeons, we are concerned that an increasing proportion of elderly patients present with MCRN. These patients pose significant clinical challenges, including advanced age, a larger burden of comorbidity [5] and potentially greater risks associated with a second colorectal resection [6].

Patients with a previous colorectal surgery may represent a high risk group who could benefit from ongoing colonoscopic surveillance. This surveillance may improve outcomes, either through polypectomy‐preventing surgery or earlier diagnosis resulting in earlier staging. This benefit becomes more important as national life expectancy improves. In New Zealand, the current mean life expectancy for someone turning 75 is 87 for males and 89 for females [7]. Some authors recommend that if life expectancy is 10 years that ongoing colonoscopic surveillance should be considered [8]. This approach is supported by the use of online tools, such as the Charlson comorbidity index [9] and the ePrognosis calculator, which estimates life expectancy by assessing overall health, age, comorbidities, and cognition [10].

The aim of our study was to document the proportion of patients undergoing colorectal resection for MCRN at age ≥ 75 and determine if the morbidity and cost of surgery in these patients were increased compared to other colorectal resections. This will help inform the appropriateness of our current surveillance policy.

## 2 Method

This is a retrospective cohort study, based on information extracted from a prospectively documented database. The inclusion criteria were acute and elective operations with resection for adenocarcinoma or an adenoma of the colon or rectum. Exclusion criteria included surgery for other malignancies (including squamous cell carcinoma or lymphoma), laparotomy without resection (including bypass), diverting stoma without resection, or resection of additional organs beyond the colon (including cystectomy, nephrectomy, hysterectomy).

The Otago Clinical Audit (OCA) database [11], which captures all surgical admissions and operations at Dunedin Hospital, was used to identify patients with elective or acute operations for colorectal neoplasia (CRN). The clinical team documents and codes all complications. The database was supplemented by the patient's electronic medical records. There were two main data extractions. To document the current incidence of surgery for MCRN ≥ 75, the first extract identified consecutive cases between January 2020 to November 2021. To assess the morbidity and cost of surgery for patients with MCRN ≥ 75, a larger group of MCRN patients was required. The second OCA extract therefore identified patients with more than one colorectal resection at Dunedin Hospital, who had their second operation between 2010 and 2020. Data extraction included (a) Patient characteristics: age, ethnicity, sex, ASA (American College of Anesthesiologists) score, Charlson Comorbidity Index; (b) Details of previous CRC diagnosis; (c) Details of the metachronous CRC presentation: acute or elective, method of diagnosis, date of last surveillance colonoscopy, preoperative anaemia; (d) Treatment and outcomes: operative treatment, duration of surgery in minutes, subsequent surgical treatment, pathological stage (TNM), postoperative complications, length of stay, readmissions, and 30 day perioperative mortality.

An estimation of cost was based on previous work where hospital stay and duration of time in theatre covered at least 80% of the total hospital costs [12, 13]. Hospital stay included time in hospital from the day of surgery to the day of discharge, and the additional number of days in hospital due to a readmission within 30 days of surgery. One day in hospital was NZD 1628.94. One minute of theatre time was NZD 65.00.

Data were summarised using standard descriptive statistics. The Shapiro–Wilk test was used to assess the distribution of data. Normally distributed data were described using mean ± standard deviation, and data not normally distributed were described by median and interquartile range. Differences in outcomes were compared using Student's *t* test for continuous normally distributed data, the Wilcoxon rank sum test for continuous non‐normally distributed data, and the Pearson's Chi‐squared test for categorical data. The Cochran‐Armitage trend test was used to compare categorical data with ordered levels. The MCRN ≥ 75 group was compared with FCRN ≥ 75 and with all other CRN with resection using unmatched data and propensity score matched data. Propensity score matching was performed using the nearest neighbour method. For comparison between MCRN ≥ 75 and FCRN ≥ 75, propensity score matching was performed for ASA score, the timing of surgery (acute v elective) and the site of resection. For comparison between MCRC ≥ 75 and all other cases, matching was for sex, hypertension, and site of surgery (chosen as this would help match predisposition to comorbidity and surgical risk which is not directly related to stopping surveillance colonoscopy). Propensity score matching and statistical tests were done using R (4.1.2) [14] with *MatchIt, dplyr*, and *DescTools* packages. Standardised mean differences were assessed. Values with *p* < 0.05 were considered statistically significant. This study was approved by the University of Otago Human Ethics Committee (HD21/081) and follows Strobe guidelines [15].

## 3 Results

Between January 2020 and November 2021, 237 patients had a resection for CRN. Of these, 18 had a previous resection for CRN (7.6%). The MCRN resection rate was 3.1% (4/130) in those < 75 and 13.1% (14/107) in those ≥ 75 years, with 78% of MCRN resections in patients ≥ 75. In the second extract, 41 patients with MCRN ≥ 75 undergoing surgery between 2010 and 2020 were identified, resulting in 278 patients. Fifty‐five with MCRN ≥ 75 years, 93 with FCRN ≥ 75 years, and 130 with CRN < 75.

Table 1 summarises the demographics, comorbidities, operative data, and pathology for the different groups. Overall, 54.3% of patients were male, although in the ≥ 75 years groups, 53% were female. The mean age for those operated on at ≥ 75 years was 82. There were fewer ASA III and IV cases in patients aged < 75 (*p* < 0.001). Rectal surgery resection with an anastomosis was more frequent in patients < 75, *p* = 0.004. Benign neoplastic disease comprised 12.9% of operations.

**TABLE 1:** Patient characteristics, comorbidity, operative data and pathology.

	MCRN ≥ 75	FCRN ≥ 75	Age < 75	FCRN ≥ 75 and Age < 75
Number	55	93	130	223
Sex (male)	23 (42)	46 (50)	82 (63)	128 (57)
Age median (IQR)	81 (75–94)	81 (75–93)	67 (27–74)	73 (27–93)
Ethnicity				
NZ European	53 (96)	91 (98)	115 (88)	205 (92)
Māori	1 (2)	1 (1)	6 (5)	7 (3)
Other	2 (4)	2 (2)	9 (7)	11 (5)
ASA				
I	1 (2)	3 (3)	11 (9)	14 (6)
II	21 (38)	45 (48)	89 (69)	134 (60)
III	32 (58)	43 (46)	30 (23)	73 (32)
IV	1 (2)	2 (2)	0	2 (1)
Hypertension	32 (58)	56 (60)	56 (43)	112 (50)
Procedure				
R hemicolectomy	25 (46)	38 (41)	38 (29)	76 (34)
High AR	2 (4)	19 (20)	36 (28)	55 (25)
Two resections	1 (2)	4 (4)	1 (1)	5 (2)
Subtotal	5 (9)	4 (4)	4 (3)	8 (4)
Other colectomy	9 (16)	3 (3)	6 (5)	9 (4)
Rectal with join	1 (2)	5 (5)	21 (16)	26 (12)
Hartmanns	6 (11)	7 (8)	2 (2)	9 (4)
APR	1 (2)	9 (10)	8 (6)	17 (8)
Transanal	5 (9)	4 (4)	14 (11)	18 (8)
Timing of surgery				
Elective	48 (87)	81 (87)	116 (89)	197 (88)
Acute	7 (13)	12 (13)	14 (11)	26 (12)
TNM stage				
Benign	8 (15)	8 (9)	20 (15)	28 (13)
I	11 (20)	12 (13)	31 (24)	43 (20)
II	19 (35)	47 (51)	20 (15)	67 (30)
III	12 (22)	18 (19)	42 (32)	60 (27)
IV	5 (9)	8 (9)	17 (13)	25 (11)

*Note:* All numbers are *n* (%) unless otherwise specified. MCRN ≥ 75: participants with metachronous colorectal neoplasia aged 75 years or more. FCRN ≥ 75: participants with a first colorectal neoplasia aged 75 years or more. All other cases = FCRN ≥ 75 + all age < 75.

Postoperative complications with and without propensity matching are summarised in Tables S1 and S2. In the MCRN ≥ 75 group, 39 (70.9%) patients developed a complication with a total of 89 complications (mean of 1.62 per patient). In the FCRN ≥ 75 group, 47 (50.5%) developed a complication with a total of 107 complications (mean 1.15). In the CRN < 75 group 59 (45.4%) developed a complication with a total of 127 complications (mean 0.98). There was no significant difference in any particular complication between groups. However, comparing the MCRN ≥ 75 and FCRN ≥ 75 groups illustrates the higher rate of complications in the MCRN ≥ 75 group was largely contributed to by ileus (27% vs. 15%), functional GI problems (Nausea, vomiting, diarrhoea, stoma problems 18% vs. 6%), UTI (11% vs. 3%), pneumonia (13% vs. 3%) and ‘other cardiac problems’ (hypotension and fluid overload, 9% vs. 2%).

Patients in the MCRN ≥ 75 group had significantly worse clinical outcomes than those in the FCRN ≥ 75 group (Table 2). Propensity matching corrected for differences in comorbidity and operations by reducing the differences in ASA III cases and high anterior resections between the two groups, giving a good match (Tables 2 and S3). The clinical differences included more complications (unmatched 70.9% vs. 50.5% *p* = 0.015 and propensity matched 70.9% vs. 51%, *p* = 0.031), readmission to hospital (unmatched 20% vs. 6.5% *p* = 0.026, propensity matched 20% vs. 5.5%, *p* = 0.045), and a longer LOS in hospital by 2 days (both 9 vs. 7 days, unmatched *p* = 0.026, propensity matched, *p* = 0.04) resulting in a more expensive hospital stay (NZD unmatched 31 000 vs. 24 100 *p* = 0.028, propensity matched 31 000 vs. 24 400 *p* = 0.074).

**TABLE 2:** Comparison of postoperative outcomes for MCRN ≥ 75, all other cases, and only FCRN ≥ 75 unmatched and with propensity matching.

Category	MCRN ≥ 75	Unmatched				Matched			
		Only FCRN ≥ 75	*p*	All other cases	*p*	Only FCRN ≥ 75	*p*	All other cases	*p*
Number	55	93		223		55		55	
Duration of procedure (min)			0.929		0.314		0.95		0.826
Median (range)	181 (15–407)	182 (16–484)		195 (16–484)		179 (16–484)		176 (39–428)	
Mean (SD)	187.3 (81.5)	192.2 (90.3)		199.7 (82.4)		195 (97)		191 (87)	
Complications	39 (71)	47 (51)	0.015	106 (48)	**0.003**	28 (51)	**0.031**	24 (44)	**0.007**
Complications per person									
Mean(SD)	1.5 (1.9)	1.3 (1.8)		1 (1.6)		1.1 (1.73)		1.3 (1.9)	
Median (range)	1 (0–12)	1 (0–9)	0.062	0 (0–10)	**0.004**	1 (0–6)	0.09	0 (0–6)	**0.021**
Mortality	4 (7)	5 (5)	0.912	6 (3)	0.219	3 (6)	1	3 (6)	1
Further surgery			0.286		0.3		0.24		**0.027**
None	49 (89)	89 (96)		198 (89)		53 (96)		47 (85)	
Acute	5 (9)	3 (3)		12 (5)		1 (2)		1 (2)	
Staged	1 (2)	1 (1)		13 (6)		1 (2)		7 (13)	
Length of Stay (days) (range)	9 (0–40)	7 (1–38)	**0.012**	6 (1–42)	**< 0.001**	7 (1–38)	**0.04**	5 (1–30)	**0.001**
Readmissions	11 (20)	6 (7)	**0.026**	12 (5)	**0.001**	3 (6)	**0.05**	3 (6)	**0.005**
Cost (NZD)			**0.028**		**0.004**				**0.045**
Median (range)	31 021.7 (1560–74 634)	24 157 (2678–92 079)		24 143 (2678–92 079)		24 403 (2678–92 079)	0.07	21 777 (5812–62 150)	
Mean (SD)	$33 291 (17 797)	$27 835 (15 379)		$26 154 (14 043)		28 287 (16 703)		24 883 (12 554)	

*Note:* All numbers are *n* (%) unless otherwise specified. MCRN ≥ 75: participants with metachronous colorectal neoplasia aged 75 years or more. FCRN ≥ 75: participants with a first colorectal neoplasia aged 75 years or more. All other cases = FCRN ≥ 75 + all age < 75. Bold values represent statistically significant result.

The MCRN ≥ 75 group was also compared to all other colorectal resections (FCRN ≥ 75 and patients with surgery aged < 75). Matching reduced differences in sex and in operative procedures (Tables 2 and S4). With younger patients in the comparison group, the differences in clinical outcomes increased, and the total number of complications became significantly different.

The impact of the surgeons' decision making when treating elderly and more comorbid patients with rectal cancer was also assessed (Table S5). In patients aged < 75 years, 21 (68%) patients had a rectal resection with an anastomosis. In those ≥ 75 years, six (27%) cases were anastomosed (There were also 10 abdominoperineal resections and six rectal Hartmann's), *p* = 0.004. The proportion of transanal excisions were similar in both groups. An analysis removing all transabdominal rectal operations with propensity matching resulted in 51 patients with MCRC ≥ 75 years being matched to 51 in the ‘all other CRN’ group (FCRN ≥ 75 + CRN < 75). In the ‘all other CRN’ group, differences were further increased, with fewer complications, readmissions, shorter LOS and a reduction in cost of approximately NZD 9000. The longer median operating time of 9 min in the MCRC ≥ 75 group was not significant.

We estimated that the cost of surgery in cases with MCRN ≥ 75 was NZD 317 777. If a colonoscopy costs approximately NZD 1250, this would cover 250 colonoscopies per annum. If surveillance colonoscopy prevented MCRN ≥ 75 by 80%, this would cover the cost of approximately 200 colonoscopies.

## 4 Discussion

The incidence of MCRN in patients ≥ 75 requiring surgery was higher than expected at 13.1%. Rates of MCRC are in the range of 2%–10% [16] and increase with time after diagnosis of the primary CRC is reported with rates of 1.8%, 3.4%, and 7.2% at 5, 10, and 20 years respectively [17]. Rates of metachronous high‐risk adenomas are 8.5%–37.2% at 5 years, depending on the number of high‐risk findings at the index colonoscopy [18]. The odds of CRN increases 1.78 per 10 years of age when comparing individuals 76–80 to those aged 50–75 [2, 19]. Overall, the high incidence of MCRN ≥ 75 in our cohort is consistent with stopping surveillance colonoscopy at an age when the development of advanced neoplasia increases.

Patients having a second bowel resection for MCRN ≥ 75 were ‘high risk’. They had more complications, longer LOS, more readmissions, and a mean increased cost of greater than NZD 5000 than matched FCRN ≥ 75 patients. These differences were further increased when compared to more routine resections (all other CRN resections) and when adjusting for changes in surgical management of rectal cancer in older patients. The main reason for the increased LOS, readmission rate, and costs was the high rate of complications, in approximately 70% of MCRN ≥ 75 patients. One possible cause is the risk of complications increasing when one of the named blood vessels to the colon has already been removed [6]. Resecting another named blood vessel may contribute to more ileus and functional bowel problems, increasing the duration of time on intravenous fluids and risk of fluid overload, pneumonia and UTI.

The most important aspect of our cost analysis is that it enabled a cost comparison to be made. This confirmed that surgery for MCRN ≥ 75 patients was significantly more expensive than for other groups. Weaknesses include our cost analysis being an approximation and that costs vary within different health care systems. Key data required for cost modelling—including the number of patients on surveillance for CRC or polyp disease, costs of diagnosis and treatment for MCRN ≥ 75 patients not having surgery, and costs for stoma appliances−were unavailable. However, our approximation of surgical costs in the MCRN ≥ 75 group suggests that this may cover the volume of colonoscopies required for ongoing surveillance of patients ≥ 75 who previously had surgery for CRN.

As MCRC surgery in those ≥ 75 is morbid for patients and costly for health providers, what can be done to decrease surgical risk in these patients? We identified that the surgeon's approach to rectal cancer in patients ≥ 75 years of age was different to those < 75 years, with more APR and rectal Hartmann's procedures and fewer anastomoses. Reasons include poor preoperative function and greater levels of comorbidity increasing the risk of a rectal anastomosis. As appropriate decision making is already demonstrated, it is unlikely that clinical decision making can be significantly improved. The key to improving outcomes is preventing MCRN ≥ 75 to avoid surgery.

While there are differences in international recommendations for colonoscopic screening [8] and/or surveillance after the age of 75, there is agreement that potential benefits need to be weighed against comorbidities. It is also recognized that colonoscopy after age 75 is more difficult because of poorer bowel preparation in up to 16% of patients aged 80 and a higher rate of bleeding, perforation, and cardiopulmonary events [8, 20]. In Australia and Europe, surveillance is offered between 75 and 80 years based on limited comorbidities as measured by a Charlson comorbidity index score [21]. The American Gastroenterology Association, the American Society of Gastrointestinal Endoscopy, and the American College of Physicians [8] recommended continuing screening colonoscopy up to the age of 85 if patient life expectancy is estimated to be 10 years. While our study does not address the optimal age for stopping surveillance, it does make three contributions. Firstly, it highlights that morbidity and cost of surgery for cancers *not prevented* because surveillance colonoscopy has been stopped should be factored into modeling of surveillance colonoscopy. Secondly, MCRN surgery in patients greater than 75 years of age is morbid and expensive and should be avoided whenever possible. Thirdly, the results suggest that patients with previous surgery for CRN should have ongoing surveillance as long as they are fit for surgery. In our study, the mean age for surgery in the MCRN ≥ 75 group was 82; in another New Zealand hospital, this was a median of 10.4 years after the last colonoscopy [22] potentially supporting surveillance for CRN at appropriate time intervals for 5–10 years depending on patient health.

This study has a number of limitations. The retrospective data limits the ability to explore potential questions that may arise. Issues around costs have already been discussed. Another limitation was that 41 of the 55 MCRC ≥ 75 patients were treated in the 10 years before the group of patients to which they were compared This raised the possibility that earlier patients may have stayed longer in hospital. However, our hospital ERAS program was established before 2010 and we noted that the increase in LOS was more closely related to a higher rate of complications than to the year of surgery.

In conclusion, the number of patients aged ≥ 75 years who present with a second neoplastic growth requiring surgery is increasing. These patients have a high risk of complications, leading to worse patient outcomes and increased health care costs. Ongoing surveillance colonoscopy for those who have had surgery for colorectal neoplasia and are well enough to proceed to surgery would be beneficial for patients and health care providers. This study also demonstrates that the high cost of unnecessary surgery when colonoscopy surveillance is stopped at 75 years is an important variable to include in modeling of future colonoscopy surveillance programs.

## Author Contributions

**Ammar Manasawala:** investigation, writing – original draft, writing – review and editing, formal analysis. **John Woodfield:** conceptualization, investigation, writing – original draft, methodology, writing – review and editing, formal analysis, supervision, resources. **Kari Clifford:** investigation, writing – original draft, methodology, writing – review and editing, formal analysis, project administration, supervision, data curation, resources. **Mark Thompson Fawcett:** conceptualization, writing – review and editing, formal analysis, supervision.

## Disclosure

The authors have nothing to report.

## Supporting information


**Table S1.** Complications in each group of patients.
**Table S2.** Complications between groups after propensity matching.
**Table S3.** Standardized mean differences after propensity matching.
**Table S4.** Comparison of patient characteristics and postoperative outcomes for MCRC ≥ 5 with CRN < 75.
**Table S5.** Differences in MCRC against all other cases (FRCN ≥ 75 + CRN < 75) after removing cases with transabdominal rectal cancer surgery ≥ 75.

## References

[1] Ministry of Health, New Zealand , “New Cancer Registrations,” 2019, https://www.health.govt.nz.

[2] I. Laish , L. Katz , S. Ben‐Horin , D. Yablecovitch , and T. Naftali , “Risk of Metachronous Neoplasia on Surveillance Colonoscopy Among Young and Older Patients After Polypectomy,” Digestive and Liver Disease 52, no. 4 (2020): 427–433. 32037272 10.1016/j.dld.2019.12.147

[3] R. L. Cali , R. M. Pitsch , A. G. Thorson , et al., “Cumulative Incidence of Metachronous Colorectal Cancer,” Diseases of the Colon and Rectum 36, no. 4 (1993): 388–393. 8458267 10.1007/BF02053945

[4] “Guidance on Surveillance for People at Increased Risk of Colorectal Cancer,” 2012.

[5] D. Papamichael , R. A. Audisio , B. Glimelius , et al., “Treatment of Colorectal Cancer in Older Patients: International Society of Geriatric Oncology (SIOG) Consensus Recommendations 2013,” Annals of Oncology 26, no. 3 (2015): 463–476. 25015334 10.1093/annonc/mdu253

[6] J. H. Lefevre , F. Bretagnol , L. Maggiori , M. Ferron , A. Alves , and Y. Panis , “Redo Surgery for Failed Colorectal or Coloanal Anastomosis: A Valuable Surgical Challenge,” Surgery 149, no. 1 (2011): 65–71. 20451231 10.1016/j.surg.2010.03.017

[7] Stats_NZ. Statistics New Zealand , “National Population Projections 2022 (Base)–2073,” 2022.

[8] J. Nee , R. Z. Chippendale , and J. D. Feuerstein , “Screening for Colon Cancer in Older Adults: Risks, Benefits, and When to Stop,” Mayo Clinic Proceedings 95, no. 1 (2020): 184–196. 31902414 10.1016/j.mayocp.2019.02.021

[9] QxMD , Charlson Comorbidity Index Score (CCI Score), (2020), https://qxmd.com/calculate/calculator_879/charlson‐comorbidity‐index‐score‐cci‐score.

[10] C. K. Suemoto , P. Ueda , H. Beltran‐Sanchez , et al., “Development and Validation of a 10‐Year Mortality Prediction Model: Meta‐Analysis of Individual Participant Data From Five Cohorts of Older Adults in Developed and Developing Countries,” Journals of Gerontology. Series A, Biological Sciences and Medical Sciences 72, no. 3 (2016): glw166. 10.1093/gerona/glw166 PMC6075520 27522061

[11] University of Otago , “Otago Clinical Audit,” https://www.otago.ac.nz/otago‐clinical‐audit/index.html.

[12] J. Woodfield , M. Hulme‐Moir , and J. Ly , “A Comparison of the Cost of Primary Closure or Rectus Abdominis Myocutaneous Flap Closure of the Perineum After Abdominoperineal Excision,” Colorectal Disease 19, no. 10 (October 2017): 934–941. 28436214 10.1111/codi.13690

[13] J. C. Woodfield , A. M. Van Rij , R. A. Pettigrew , A. Van Der Linden , and D. Bolt , “Using Cost of Infection as a Tool to Demonstrate a Difference in Prophylactic Antibiotic Efficacy: A Prospective Randomized Comparison of the Pharmacoecomonic Effectiveness of Ceftriaxone and Cefotaxime Prophylaxis in Abdominal Surgery,” World Journal of Surgery 29 (2024): 18–24. 10.1007/s00268-004-7257-z 15599747

[14] R Core T , R: A Language and Environment for Statistical Computing (R Foundation for Statistical Computing, 2021).

[15] E. von Elm , D. Altman , M. Egger , et al., “The Strengthening the Reporting of Observational Studies in Epidemiology (STROBE)statement: Guidelines for Reporting Observational Studies,” Journal of Clinical Epidemiology 61, no. 4 (2008): 344–349. 18313558 10.1016/j.jclinepi.2007.11.008

[16] Y. Zhang , A. Karahalios , Y. K. Aung , A. K. Win , A. Boussioutas , and M. A. Jenkins , “Risk Factors for Metachronous Colorectal Cancer and Advanced Neoplasia Following Primary Colorectal Cancer: A Systematic Review and Meta‐Analysis,” BMC Gastroenterology 23, no. 1 (2023): 421. 38036994 10.1186/s12876-023-03053-2 PMC10688466

[17] A. M. Bouvier , M. Latournerie , V. Jooste , C. Lepage , V. Cottet , and J. Faivre , “The Lifelong Risk of Metachronous Colorectal Cancer Justifies Long‐Term Colonoscopic Follow‐Up,” European Journal of Cancer 44, no. 4 (2008): 522–527. 18255278 10.1016/j.ejca.2008.01.007

[18] H. R. Yun , L. J. Lee , J. H. Park , et al., “Local Recurrence After Curative Resection in Patients With Colon and Rectal Cancers,” International Journal of Colorectal Disease 23, no. 11 (2008): 1081–1087. 18688621 10.1007/s00384-008-0530-0

[19] H. Strul , R. Kariv , M. Leshno , et al., “The Prevalence Rate and Anatomic Location of Colorectal Adenoma and Cancer Detected by Colonoscopy in Average‐Risk Individuals Aged 40–80 Years,” Official Journal of the American College of Gastroenterology|ACG 101, no. 2 (2006): 255–262, 10.1111/j.1572-0241.2006.00430.x. 16454827

[20] L. W. Day , A. Kwon , J. M. Inadomi , L. C. Walter , and M. Somsouk , “Adverse Events in Older Patients Undergoing Colonoscopy: A Systematic Review and Meta‐Analysis,” Gastrointestinal Endoscopy 74, no. 4 (2011): 885–896. 21951478 10.1016/j.gie.2011.06.023 PMC3371336

[21] H. Giulio , J.‐M. Dumonceau , J. Regula , et al., “Post‐Polypectomy Colonoscopy Surveillance: European Society of Gastrointestinal Endoscopy (ESGE) Guideline—Update 2020,” Endoscopy 52, no. 8 (2020): 687–700. 32572858 10.1055/a-1185-3109

[22] M. J. McGuinness , N. Joseph , S. J. G. Richards , and J. M. Speight , “A Retrospective Study of Colonoscopic Surveillance in the Elderly,” ANZ Journal of Surgery 93, no. 9 (September 2023): 2138–2142. 36811312 10.1111/ans.18344

