# Supplementary material for: The Impact of Metachronous Colorectal Neoplasia Requiring Surgery After Cessation of Colonoscopic Surveillance at Age 75
Source: ANZ J Surg. 2025 May 30;95(9):1793–9. doi: 10.1111/ans.70199 (PMC12484390; doi:10.1111/ans.70199)
Supplement: Supplementary file 1 — Table S1. Complications in each group of patients. Table S2. Complications between groups after propensity matching. Table S3. Standardized mean differences after propensity matching. Table S4. Comparison of patient characteristics and postoperative outcomes for MCRC ≥ 5 with CRN < 75. Table S5. Differences in MCRC against all other cases (FRCN ≥ 75 + CRN < 75) after removing cases with transabdominal rectal cancer surgery ≥ 75. [file ANS-95-1793-s001.docx]

**SUPPLEMENT**

**SECTION A: FREQENCY OF COMPLICATIONS**

**Table S1: Complications in each group of patients**

|  | **MCRN≥ 75** | **FRCN≥ 75** | **CRN<75** | **Total** |
| --- | --- | --- | --- | --- |
| Number of patients | 55 | 93 | 130 | 278 |
| Patients with complications | 39 (70.9%) | 47 (50.5%) | 59 (45.4%) | 145 (52.2%) |
| Total number of complications (ratio) | 89 (1.62) | 107 (1.15) | 127 (0.98) | 323 (1.16) |
| 30 day mortality | 4 (7.3%) | 5 (5.4%) | 1 (0.8%) | 10 (3.6%) |
| **Gastrointestinal** |  |  |  |  |
| Ileus | 15 (27.3%) | 14 (15.1%) | 24 (18.5%) | 53 (19.1%) |
| Anastomotic leak | 3 (5.5%) | 7 (7,5%) | 7 (5.4%) | 17 (6.1%) |
| Small bowel obstruction | 2 (3.6%) | 1 (1.1%( | 1 (0.8%) | 4 (1.4%) |
| Other | 10 (18.2%) | 6 (6.5%) | 8 (6.1%) | 24 (8.6%) |
| **Urinary/Renal** |  |  |  |  |
| UTI | 6 (10.9%) | 3 (3.2%) | 7 (5.4%) | 16 (5.8%) |
| Urinary retention | 3 (5.5%) | 9 (9.7%) | 6 (4.6%) | 18 (6.5%) |
| Acute kidney injury | 3 (5.5%) | 5 (5.4%) | 4 (3.1%) | 12 (4.3%) |
| **Wound** |  |  |  |  |
| Incisional SSI | 2 (3.6%) | 5 (5.4%) | 5 (3.8%) | 12 (4.3%) |
| Small bowel herniation | 1 (1.8%) | 0 (0.0%) | 0 (0.0%) | 1 (0.4%) |
| **Pulmonary** |  |  |  |  |
| Pneumonia | 7 (12.7%) | 3 (3.2%) | 2 (1.5%) | 12 (4.3%) |
| Other | 1 (1.8%) | 3 (3.2%) | 2 (1.5%) | 6 (2.2%) |
| **Infective (other)** |  |  |  |  |
| Fever | 1 (1.8%) | 7 (7.5%) | 18 (13.8%) | 26 (9.4%) |
| Sepsis | 1 (1.8%) | 2 (2.2%) | 4 (3.1%) | 7 (2.5%) |
| Space SSI | 1 (1.8%) | 4 (4.3%) | 4 (3.1%) | 9 (3.2%) |
| Other | 5 (9.1%) | 3 (3.2%) | 1 (0.8%) | 9 (3.2%) |
| **Haematological** |  |  |  |  |
| Bleeding | 2 (3.6%) | 6 (6.5%) | 1 (0.8%) | 9 (3.2%) |
| DVT and PE | 2 (3.6%) | 0 (0.0%) | 1 (0.8%) | 3 (1.1%) |
| Other | 2 (3.6%) | 4 (4.3%) | 2 (1.5%) | 8 (2.9%) |
| **Cardiac** |  |  |  |  |
| Atrial fibrillation | 3 (5.5%) | 5 (5.4%) | 7 (5.4%) | 15 (5.4%) |
| Other arrythmia | 1 (1.8%) | 3 (3.2%) | 4 (3.1%) | 8 (2.9%) |
| Heart failure | 2 (3.6%) | 1 (1.1%) | 1 (0.8%) | 4 (1.4%) |
| STEMI | 1 (1.8%) | 0 (0.0%) | 0 (0.0%) | 1 (0.4%) |
| Other | 5 (9.1%) | 2 (2.2%) | 1 (0.8%) | 8 (2.9%) |
| **Neurological** |  |  |  |  |
| Post-op confusion | 3 (5.5%) | 1 (1.1%) | 5 (3.8%) | 9 (3.2%) |
| Cerebrovascular accident | 1 (1.8%) | 0 (0.0%) | 1 (0.8%) | 2 (0.7%) |
| **Metabolic** |  |  |  |  |
| Electrolyte and pH | 3 (5.4%) | 7 (7.6%) | 6 (4.6%) | 16 (5.8%) |
| **Other** |  |  |  |  |
| All other complications | 3 (5.5%) | 6 (6.5%) | 5 (3.8%) | 14 (5.0%) |

UTI: urinary tract infection

SSI: Surgical site infection

DVT: Deep vein thrombosis

PE: pulmonary embolism

**Table S2 Complications between groups after propensity matching**

|  | MRCN≥ 75 | FRCN≥ 75 | CRN<75 | Total |
| --- | --- | --- | --- | --- |
| Number of patients | 55 | 55 | 55 | 165 |
| Number of patients with a Complication | 39 (70.9%) | 29 (52.7%) | 28 (50.9%) | 96 (58.2%) |
| Total number of complications | 89 (1.62) | 62 (1.127) | 55 (1.00) | 206 (1.248) |
| 30 day mortality | 4 (7.3%) | 3 (5.5%) | 0 | 7 (4.2%) |
| **Gastrointestinal** |  |  |  |  |
| Ileus | 15 (27.3%) | 9 (16.4%) | 15 (27.3%) | 39 (23.6%) |
| Anastomotic leak | 3 (5.5%) | 3 (5.5%) | 1 (1.8%) | 7 (4.2%) |
| Small bowel obstruction | 2 (3.6%) | 1 (1.8%) | 0 | 3 (1.8%) |
| Other | 10 (18.2%) | 2 (3.6%) | 6 (10.9%) | 21 (7.3%) |
| **Urinary/Renal** |  |  |  |  |
| UTI | 6 (10.9%) | 1 (1.8%) | 2 (3.6%) | 9 (5.5%) |
| Urinary retention | 3 (5.5%) | 6 (10.9%) | 0 | 9 (5.5%) |
| Acute kidney injury | 3 (5.5%) | 1 (1.8%) | 1 (1.8%) | 5 (3.0%) |
| **Wound** |  |  |  |  |
| Incisional SSI | 2 (3.6%) | 5 (9.1%) | 3 (5.5%) | 10 (6.1%) |
| Small bowel herniation | 1 (1.8%) | 0 | 0 | 1 (0.6%) |
| **Pulmonary** |  |  |  |  |
| Pneumonia | 7 (12.7%) | 2 (3.6%) | 1 (1.8%) | 10 (6.1%) |
| Other | 1 (1.8%) | 2 (3.6%) | 1 (1.8%) | 4 (2.4%) |
| **Infective (other)** |  |  |  |  |
| Fever | 1 (1.8%) | 6 (10.9%) | 9 (16.4%) | 16 (9.7%) |
| Sepsis | 1 (1.8%) | 2 (3.6%) | 2 (3.6%) | 5 (3.0%) |
| Space SSI | 1 (1.8%) | 2 (3.6%) | 0 | 3 (1.8%) |
| Other | 5 (9.1%) | 1 (1.8%) | 0 | 6 (3.6%) |
| **Haematological** |  |  |  |  |
| Bleeding | 2 (3.6%) | 3 (5.5%) | 0 | 5 (3.0%) |
| DVT and PE | 2 (3.6%) | 0 | 1 (1.8%) | 3 (1.8%) |
| Other | 2 (3.6%) | 3 (5.5%) | 2 (3.6%) | 7 (4.2%) |
| **Cardiac** |  |  |  |  |
| Atrial fibrillation | 3 (5.5%) | 3 (5.5%) | 2 (3.6%) | 8 (4.8%) |
| Other arrythmia | 1 (1.8%) | 2 (3.6%) | 3 (5.5%) | 6 (3.6%) |
| Heart failure | 2 (3.6%) | 1 (1.8%) | 0 | 3 (1.8%) |
| STEMI | 1 (1.8%) | 0 | 0 | 1 (0.6%) |
| Other | 5 (9.1%) | 0 | 0 | 5 (3.0%) |
| **Neurological** |  |  |  |  |
| Post-op confusion | 3 (5.5%) | 2 (3.6%) | 1 (1.8%) | 6 (3.6%) |
| Cerebrovascular accident | 1 (1.8%) | 0 | 0 | 1 (0.6%) |
| **Metabolic** |  |  |  |  |
| electrolyte and pH disturbances | 3 (5.4%) | 2 (3.6%) | 3 (5.5%) | 8 (4.8%) |
| **Other** |  |  |  |  |
| All other complications | 3 (5.5%) | 3 (5.5%) | 1 (1.8%) | 7 (4.2%) |

UTI: urinary tract infection

SSI: Surgical site infection

DVT: Deep vein thrombosis

PE: pulmonary embolism

**SECTION B: Quality of propensity matching**

**Table S3 Standardized mean differences after propensity matching**

Standardised mean differences between MCRN≥ 75 and matched groups:

|  | FCRN≥ 75 | CRN<75 | All others |
| --- | --- | --- | --- |
| Sex | 0.14645 | 0.217815 | 0.036649 |
| Age | 0.018993 | 2.346365 | 0.893804 |
| ASA | 0.223861 | 0.600216 | 0.306782 |
| Hypertension | 0.074278 | 0.108908 | 0.072703 |
| Procedure | 0.110624 | 0.120154 | 0.027579 |
| Pathology | 0.174488 | 0.085088 | 0.193359 |
| Timing of surgery | 0.052516 | 0.052516 | 0.149948 |

Standardised mean differences between MCRN≥ 75 and matched groups with rectal cases removed:

|  | FCRN≥ 75 | CRN<75 | All others |
| --- | --- | --- | --- |
| Sex | 0.040357 | 0.276358 | 0.158297 |
| Age | 0.00404 | 2.152048 | 0.896662 |
| ASA | 0.036723 | 0.567976 | 0.181747 |
| Hypertension | 0.040357 | 0.235757 | 0.080378 |
| Procedure | 0.245438 | 0.259166 | 0.055902 |
| Pathology | 0.167725 | 0.030252 | 0.244433 |
| Timing of surgery | 0.054839 | 0 | 0 |

FCRN ≥75: participants with a first colorectal neoplasia aged 75 years or more

CRN <75: participants with a colorectal neoplasia aged less than 75 years

Interpretation: generally, an SMD of 0.2 is considered small, 0.5 is medium, and 0.8 is large, according to Cohen's conventional interpretation

**SECTION C: Additional data on comparison between groups with and without propensity matching**

**Table S4 Comparison of patient characteristics and postoperative outcomes for MCRC ≥ 75 with CRN<75**

| **Category** | **Unmatched** | | | **Matched** | | | |  |
| --- | --- | --- | --- | --- | --- | --- | --- | --- |
|  | **MCRN≥ 75** | **CRN<75** |  | **MCRN≥ 75** | **CRN<75** |  |  |  |
| Number | 55 | 130 |  | 55 |  |  |  |  |
| Sex | 23 (41.8%) | 82(63.1%) |  | 23 (41.8%) | 29 (52.7%) |  |  |  |
| Age Median (IQR) | 81 (75-94) | 67 (27-74) |  | 81 (75-94) | 68 (29-74) |  |  |  |
| ASA |  |  |  |  |  |  |  |  |
| I | 1 (1.8% ) | \| 11 \| ( \| 8.5% \| ) \| \| --- \| --- \| --- \| --- \| |  | 1 (1.8% ) | 2 (3.6%) |  |  |  |
| II | \| 21 \| ( \| 38.2% \| ) \| \| --- \| --- \| --- \| --- \| | \| 89 \| ( \| 68.5% \| ) \| \| --- \| --- \| --- \| --- \| |  | \| 21 \| ( \| 38.2% \| ) \| \| --- \| --- \| --- \| --- \| | 36 (65.5%) |  |  |  |
| III | \| 32 \| ( \| 58.2% \| ) \| \| --- \| --- \| --- \| --- \| | \| 30 \| ( \| 23.1% \| ) \| \| --- \| --- \| --- \| --- \| |  | \| 32 \| ( \| 58.2% \| ) \| \| --- \| --- \| --- \| --- \| | 17 (30.9%) |  |  |  |
| IV | \| 1 \| ( \| 1.8% \| ) \| \| --- \| --- \| --- \| --- \| | 0 |  | \| 1 \| ( \| 1.8% \| ) \| \| --- \| --- \| --- \| --- \| | 0 |  |  |  |
| Hypertension | \| 32 \| ( \| 58.2% \| ) \| \| --- \| --- \| --- \| --- \| | \| 56 \| ( \| 43.1% \| ) \| \| --- \| --- \| --- \| --- \| |  | \| 32 \| ( \| 58.2% \| ) \| \| --- \| --- \| --- \| --- \| | 26 (47.3%) |  |  |  |
| Procedure |  |  |  |  |  |  |  |  |
| R hemicolectomy | \| 25 \| ( \| 45.5% \| ) \| \| --- \| --- \| --- \| --- \| | \| 38 \| ( \| 29.2% \| ) \| \| --- \| --- \| --- \| --- \| |  | \| 25 \| ( \| 45.5% \| ) \| \| --- \| --- \| --- \| --- \| | 21 (38.2%) |  |  |  |
| High AR | \| 2 \| ( \| 3.6% \| ) \| \| --- \| --- \| --- \| --- \| | 36 (27.7%) |  | \| 2 \| ( \| 3.6% \| ) \| \| --- \| --- \| --- \| --- \| | 2 (3.6%) |  |  |  |
| Double | \| 1 \| ( \| 1.8% \| ) \| \| --- \| --- \| --- \| --- \| | \| 1 \| ( \| 0.8% \| ) \| \| --- \| --- \| --- \| --- \| |  | \| 1 \| ( \| 1.8% \| ) \| \| --- \| --- \| --- \| --- \| | 0 |  |  |  |
| Subtotal | \| 5 \| ( \| 9.1% \| ) \| \| --- \| --- \| --- \| --- \| | \| 4 \| ( \| 3.1% \| ) \| \| --- \| --- \| --- \| --- \| |  | \| 5 \| ( \| 9.1% \| ) \| \| --- \| --- \| --- \| --- \| | 3 (5.5%) |  |  |  |
| Other colectomy | \| 9 \| ( \| 16.4% \| ) \| \| --- \| --- \| --- \| --- \| | \| 6 \| ( \| 4.6% \| ) \| \| --- \| --- \| --- \| --- \| |  | \| 9 \| ( \| 16.4% \| ) \| \| --- \| --- \| --- \| --- \| | 5 (9.1%) |  |  |  |
| Hartmanns | \| 6 \| ( \| 10.9% \| ) \| \| --- \| --- \| --- \| --- \| | \| 2 \| ( \| 1.5% \| ) \| \| --- \| --- \| --- \| --- \| |  | \| 6 \| ( \| 10.9% \| ) \| \| --- \| --- \| --- \| --- \| | 2 (3.6%) |  |  |  |
| Rectal with join | \| 1 \| ( \| 1.8% \| ) \| \| --- \| --- \| --- \| --- \| | \| 21 \| ( \| 16.2% \| ) \| \| --- \| --- \| --- \| --- \| |  | \| 1 \| ( \| 1.8% \| ) \| \| --- \| --- \| --- \| --- \| | 11 (20%) |  |  |  |
| APR | \| 1 \| ( \| 1.8% \| ) \| \| --- \| --- \| --- \| --- \| | \| 8 \| ( \| 6.2% \| ) \| \| --- \| --- \| --- \| --- \| |  | \| 1 \| ( \| 1.8% \| ) \| \| --- \| --- \| --- \| --- \| | 2 (3.6%) |  |  |  |
| Transanal | 5(9.1%) | \| 14 \| ( \| 10.8% \| ) \| \| --- \| --- \| --- \| --- \| |  | 5(9.1%) | 4 (7.3%) |  |  |  |
| Pathology |  |  |  |  |  | 0.653 |  |  |
| I | 11 (20.0%) | \| 31 \| ( \| 23.8% \| ) \| \| --- \| --- \| --- \| --- \| |  | 11 (20.0%) | 12 (22%) |  |  |  |
| II | 19 (34.5%) | \| 20 \| ( \| 15.4% \| ) \| \| --- \| --- \| --- \| --- \| |  | 19 (34.5%) | 9 (16%) |  |  |  |
| III | 12 (21.8%) | \| 42 \| ( \| 32.3% \| ) \| \| --- \| --- \| --- \| --- \| |  | 12 (21.8%) | 15 (27%) |  |  |  |
| IV | 5 (9.1%) | \| 31 \| ( \| 23.8% \| ) \| \| --- \| --- \| --- \| --- \| |  | 5 (9.1%) | 9 (16%) |  |  |  |
| Timing of Surgery |  |  |  |  |  |  |  |  |
| Elective | \| 48 \| ( \| 87.3%) \| ) \| \| --- \| --- \| --- \| --- \| | \| 116 \| ( \| 89.2%) \| ) \| \| --- \| --- \| --- \| --- \| |  | \| 48 \| ( \| 87.3%) \| ) \| \| --- \| --- \| --- \| --- \| | 47 (85.5%) |  |  |  |
| Acute | \| 7 \| ( \| 12.7% \| ) \| \| --- \| --- \| --- \| --- \| | \| 14 \| ( \| 10.8% \| ) \| \| --- \| --- \| --- \| --- \| |  | \| 7 \| ( \| 12.7% \| ) \| \| --- \| --- \| --- \| --- \| | 8 (14.5%) |  |  |  |
| **Clinical outcomes** | | | | | | | | |
|  |  |  | P |  |  | P |  |  |
| Duration of surgery | |  | 0.174 |  |  | 0.048 |  |  |
| Median | 181 (15-407) | 209 (28-428) |  | 181 (15-407) | 209 (29-428) |  |  |  |
| Mean | 187.3 (81.5) | 205.1 (80.1) |  | 187.3 (81.5) | 218 (79) |  |  |  |
| Patient with complications | 39 (70.9%) | 59 (45.4%) | **0.003** | 39 (70.9%) | 28 (51%) | 0.051 |  |  |
| Number of complications | 1 (0-12) | 0 (0-10) | **0.002** | 1 (0-12) | 1 (0-5) | **0.022** |  |  |
| Mortality | 4 (7.3%) |  |  | 4 (7.3%) | 0 (0%) | 0.127 |  |  |
| Further surgery | |  | 0.184 |  |  | **0.032** |  |  |
| None | 49 (89.1%) | 109 (83.8%) |  | 49 (89.1%) | 45 (82%) |  |  |  |
| Acute | 5 (9.1%) | 9 (6.9%) |  | 5 (9.1%) | 2 (3.6%) |  |  |  |
| Staged | 1 (1.8%) | 12 (9.2%) |  | 1 (1.8%) | 8 (15%) |  |  |  |
| LOS | 9 (0-40) | 5 (1-42) | **<0.001** | 9 (0-40) | 7 (1-25) | **0.004** |  |  |
| Readmissions | 11 (20.0%) | 6 (4.6%) | **0.002** | 11 (20.0%) | 2 (3.6%) | **0.018** |  |  |
| Cost |  |  | 0.0025 |  |  |  |  |  |
| Median (range) | $31,021.7 (1,560-74,634) | $23,851 (3,458-79,470) |  | $31,021.7 (1,560-74,634) | 27,772 (3,523-56,624) | **0.068** |  |  |
| Mean (sd) | $33,291 (17,797) | $24,952 (12,932) |  | $33,291 (17,797) | 26,816 (11,693) |  |  |  |

All numbers are n(%) unless otherwise specified

LOS: Length of Hospital Stay

AR: Anterior resection

APR: Abdominoperineal Resection

MCRN ≥75: participants with metachronous colorectal neoplasia aged 75 years or more

CRN <75: participants with a colorectal neoplasia aged less than 75 years

**Table S5: Differences in MCRC against all other cases (FRCN≥75+CRN<75) after removing cases with transabdominal rectal cancer surgery≥ 75.**

|  | Patient and procedural characteristics | | |
| --- | --- | --- | --- |
|  | MCRC≥ 75 | All other cases | p value |
| Number | 51 | 51 |  |
| Sex (male) | \| 19 \| ( \| 37.3% \| ) \| \| --- \| --- \| --- \| --- \| | \| 23 \| ( \| 45.1% \| ) \| \| --- \| --- \| --- \| --- \| |  |
| Age Median (IQR) | 81 (75-94) | 74 (29-93) |  |
| ASA |  |  |  |
| I | \| 1 \| ( \| 2.0% \| ) \| \| --- \| --- \| --- \| --- \| | 0 |  |
| II | \| 20 \| ( \| 39.2% \| ) \| \| --- \| --- \| --- \| --- \| | \| 26 \| ( \| 51.0% \| ) \| \| --- \| --- \| --- \| --- \| |  |
| III | \| 29 \| ( \| 56.9% \| ) \| \| --- \| --- \| --- \| --- \| | 25(49.0%) |  |
| IV | \| 1 \| ( \| 2.0% \| ) \| \| --- \| --- \| --- \| --- \| | 0 |  |
| Hypertension | \| 31 \| ( \| 60.8% \| ) \| \| --- \| --- \| --- \| --- \| | 33 (64.7%) |  |
| Procedure |  |  |  |
| R hemicolectomy | \| 25 \| ( \| 49.0% \| ) \| \| --- \| --- \| --- \| --- \| | \| 27 \| ( \| 52.9% \| ) \| \| --- \| --- \| --- \| --- \| |  |
| Other colectomy | \| 9 \| ( \| 17.6% \| ) \| \| --- \| --- \| --- \| --- \| | \| 8 \| ( \| 15.7% \| ) \| \| --- \| --- \| --- \| --- \| |  |
| Subtotal | \| 5 \| ( \| 9.8% \| ) \| \| --- \| --- \| --- \| --- \| | \| 4 \| ( \| 7.8% \| ) \| \| --- \| --- \| --- \| --- \| |  |
| Double | \| 1 \| ( \| 2.0% \| ) \| \| --- \| --- \| --- \| --- \| | \| 1 \| ( \| 2.0% \| ) \| \| --- \| --- \| --- \| --- \| |  |
| High AR | \| 2 \| ( \| 3.9% \| ) \| \| --- \| --- \| --- \| --- \| | \| 2 \| ( \| 3.9% \| ) \| \| --- \| --- \| --- \| --- \| |  |
| Hartmanns | \| 4 \| ( \| 7.8% \| ) \| \| --- \| --- \| --- \| --- \| | \| 3 \| ( \| 5.9% \| ) \| \| --- \| --- \| --- \| --- \| |  |
| Rectal with join | 0 | 0 |  |
| APR | 0 | 0 |  |
| Transanal | \| 5 \| ( \| 9.8% \| ) \| \| --- \| --- \| --- \| --- \| | \| 6 \| ( \| 11.8% \| ) \| \| --- \| --- \| --- \| --- \| |  |
| Pathology |  |  |  |
| I | 11 (22%) | 9 (18%) |  |
| II | 17 (33%) | 16 (31%) |  |
| III | 11 (22%) | 12 (24%) |  |
| IV | 4 (7.8%) | 8 (16%) |  |
| Timing of Surgery |  |  |  |
| Elective | \| 44 \| ( \| 86.3% \| ) \| \| --- \| --- \| --- \| --- \| | \| 44 \| ( \| 86.3% \| ) \| \| --- \| --- \| --- \| --- \| |  |
| Acute | 7 (13.7%) | \| 7 \| ( \| 13.7% \| ) \| \| --- \| --- \| --- \| --- \| |  |
| Clinical outcomes | | | |
| Duration |  |  |  |
| Median (range) | 170 (15-407) | 161 (39-415) | 0.844 |
| Mean(sd) | 180 (78) | 177 (79) |  |
| Patient with complications | 36 (71%) | 20 (39%) | **0.003** |
| Number of complications | 1 (0-12) | 0 (0-5) | 0.015 |
| Mortality | 4 (7.8%) | 3 (5.9%) | 1.000 |
| Further surgery |  |  | **0.107** |
| None | 45 (88%) | 46 (90%) |  |
| Acute | 5 (9.8%) | 1 (2.0%) |  |
| Staged | 1 (2.0%) | 4 (7.8%) |  |
| LOS | 9 (0-40) | 5 (1-25) | **<0.001** |
| Readmissions | 10 (20%) | 1 (2.0%) | **0.011** |
| Cost |  |  | **0.001** |
| Median(range) | 29,072 (1,560-74,634) | 20,308 (5,343-56,624) |  |
| Mean (sd) | 32,924 (18,416) | 22,123 (10,328) |  |

All numbers are n(%) unless otherwise specified

LOS: Length of Hospital Stay

AR: Anterior resection

APR: Abdominoperineal Resection

MCRN ≥75: participants with metachronous colorectal neoplasia aged 75 years or more

FCRN ≥75: participants with a first colorectal neoplasia aged 75 years or more
